# The Regulation of Growth in Developing, Homeostatic, and Regenerating Tetrapod Limbs: A Minireview

**DOI:** 10.3389/fcell.2021.768505

**Published:** 2022-01-03

**Authors:** Kaylee M. Wells, Mary Baumel, Catherine D. McCusker

**Affiliations:** Department of Biology, College of Science and Mathematics, University of Massachusetts Boston, Boston, MA, United States

**Keywords:** limb development, limb regeneration, patterning, long bone growth, growth regulation

## Abstract

The size and shape of the tetrapod limb play central roles in their functionality and the overall physiology of the organism. In this minireview we will discuss observations on mutant animal models and humans, which show that the growth and final size of the limb is most impacted by factors that regulate either limb bud patterning or the elongation of the long bones. We will also apply the lessons that have been learned from embryos to how growth could be regulated in regenerating limb structures and outline the challenges that are unique to regenerating animals.

## Introduction

Although the underlying anatomy is shared, the scale and shape of limbs vary greatly among tetrapod species. The batwing is optimized for flying, horse legs are optimized for running, and snake legs have all but disappeared to allow for the serpentine movements of the body. Beyond the various impacts on locomotive abilities, limb sizing also plays key roles in activities such as eating, mating, and communication. Thus, the development of limbs that are the proportionally appropriate size for each species is essential for the functionality of these structures and the overall physiology of these animals. This review will focus on the molecular mechanisms that regulate limb growth, which will ultimately impact the overall size and functionality of the limb structures that form.

Limb formation in all tetrapod species begins with the development of a structure known as the limb bud. The limb bud is composed of an ectodermal signaling center that covers a cluster of mesodermal cells which will proliferate, pattern, and differentiate into the tissues that compose the basic blueprint of the tetrapod limb. Therefore, alterations that impact limb development, such as those involved in pattern formation and physiology in the limb bud cells, will greatly impact subsequent steps that also influence limb length. As the limb tissues continue to mature, the limb elongates through the growth of the long bones to the length that is uniquely appropriate to the body size in each species. The process by which the limb grows in relation to the rest of the organism’s body is called ontogenetic allometric growth, and alterations to this growth can greatly impact the size and functionality of the limbs.

**FIGURE 1:**
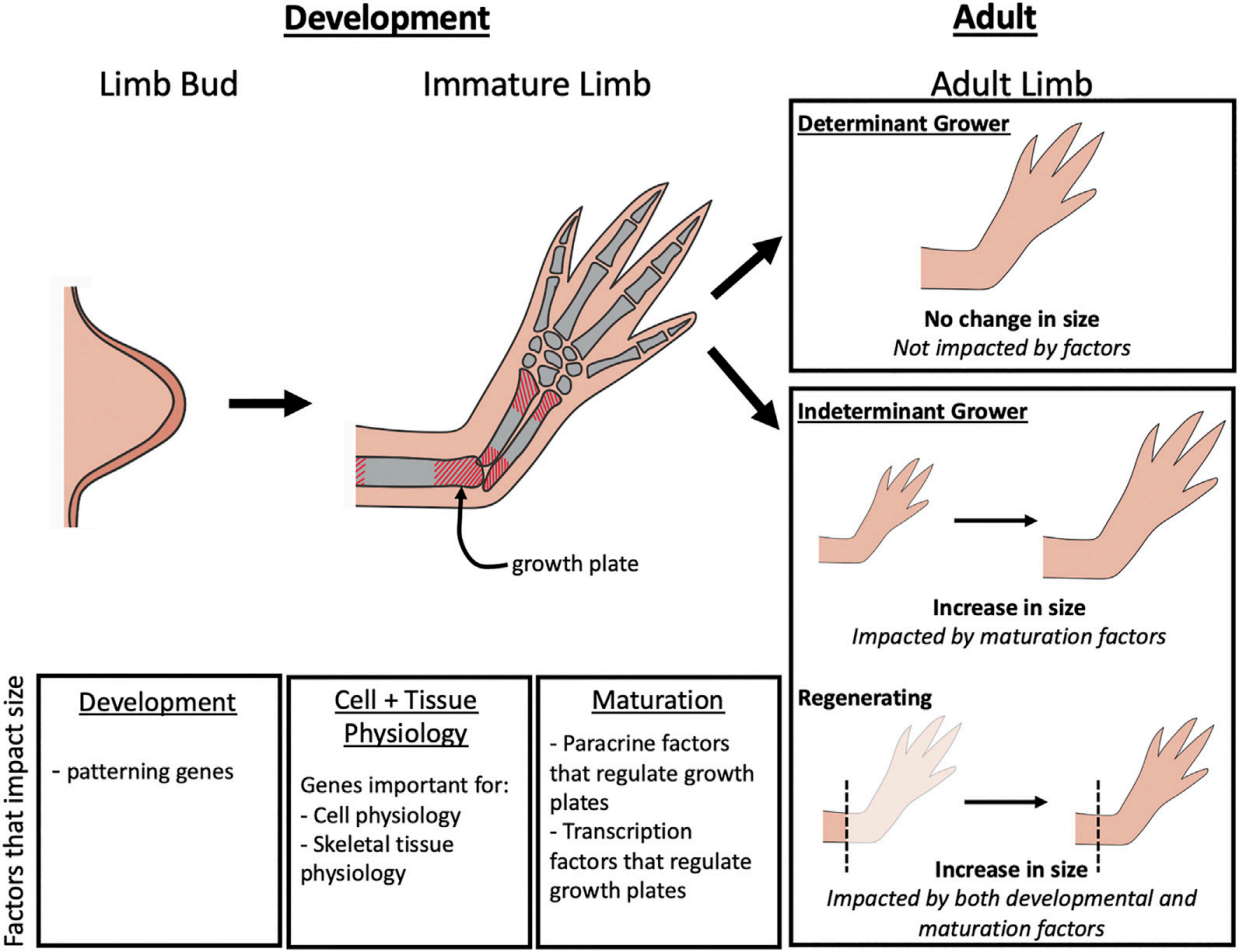
Factors that impact limb growth. During limb bud development, changes in limb patterning genes can lead to differences in the overall size of the adult limb. As the immature limb elongates, size is controlled by paracrine factors and transcription factors that regulate growth of the growth plates. In adult limbs, the factors that influence size is dependent on the type of organismal growth type (determinant or indeterminant) and whether regeneration is occurring. Limb size on determinant growers will not be impacted by regulation during adulthood, but in indeterminant growers, limb size can be impacted by maturation factors that alter growth plate activity. During regeneration, both development and maturation factors can influence limb size.

Although the mechanisms regulating limb growth are not fully elucidated, studies on developing embryonic limbs in model organisms as well as genetic characterization of humans with limb length pathologies, indicate that factors that regulate limb bud development, cell and tissue physiology, and the activity of the growth plates in the limb long bones all play important roles (Figure 1). The impact that the alteration of these different factors can have on limb size varies depending on the stage of development and whether the animal is a determinant or indeterminately growing species (Figure 1). Determinant species cease growing once they reach adulthood, whereases indeterminant species continue to grow throughout their lifecycle. Some indeterminant tetrapods, such as Urodele amphibians, retain the ability to regenerate complete limbs through adulthood, and thus require specialized regulation of the regenerating structure. In this review we will discuss the various molecular factors that contribute to limb growth (Table 1). Because most of the studies that have identified these factors were performed in mammals and birds, the focus will be on determinant species. We will then draw parallels with what is known about the mechanisms that regulate sizing during limb regeneration in Urodeles.

**TABLE 1:** Limb length phenotypes in human, mouse, and chicken.

Pathway/Topic		Mutations effect on pathway	Molecules	Limb length phenotype	References
Paracrine Factors					
IHH	Inhibitory		IHH; JAWS; SMO; EVC; EVC2; WDR11; GLI2; FOXC1	Short Limb	[1]–[11]
	Activating		IHH	Long Limb	[12]
SHH	Inhibitory		GLI3; GAS1; HAND2; ICK; DYRK2; CCD/DSH	Short Limb	[13]–[20]
FGF	Activating		VPS25; FGFR3	Short Limb	[21]–[25]
	Inhibitory		FGFR3	Long Limb	[26], [27]
			FGF8; SP8/mBtd; ERSP1/ERSP2	Short Limb	[28]–[30]
BMP	Activating		Noggin; Cerebus-like	Short Limb	[31]–[33]
			BMP2; BMP4	Long Limb	[34]
	Inhibitory		BMPR1; GDF5; ARSB; MSX1; MSX2; CDC42; PLZF; CHST11	Short Limb	[35]–[42]
TGFB	Activating		TGFB1; SKI	Long Limb	[43]–[45]
			FBN1	Short Limb	[46]
				Long Limb	[47]
Natriuretic Peptide	Activating		NPR2	Long Limb	[48]–[50]
	Inhibitory		NPPC; NPR2	Short Limb	[51]–[56]
NFKB	Activating		Chuk/IKK1	Short Limb	[57]
	Inhibitory		RGS10; RIP4; RANK/TNFRSF11 A	Short Limb	[58]–[61]
WNT	Activating		SFRP1; SFRP2; WNT4	Short Limb	[62], [63]
	Inhibitory		WNT5a; LRP6; CTNNB1; PORCN: ROR2; ROR Receptors; Prickle; RSPO2/RSPO3; RYK; WLS	Short Limb	[64]–[79]
Parathyroid Hormone	Activating		PTHrP; PTH1R (receptor)	Short Limb	[80]–[83]
	Inhibitory		PTH; PTHrP Receptor; G(s)-alpha	Short Limb	[84]–[91]
Thyroid Hormone	Inhibitory		PAX8; Trip11/GMAP210; TR-alpha; TR-beta	Short Limb	[92]–[99]
Transcriptional Modifiers					
Homeobox	Activating		PRRX1	Long Limb	[100]
	Inhibitory		HoxA13; HoxD12; HoxD13; EVX2	Short Limb	[101]–[103]
Hippo Pathway (YAP/TAZ)	Activating		MST1; MST2	Short Limb	[104]
SOX	Activating		Sox9	Long Limb	[105]
	Inhibitory		Sox5; Sox6; Sox9; Kindlin-2	Short Limb	[106]–[110]
RUNX1/2	Activating		Twist1	Short Limb	[111], [112]
	Inhibitory		CBFA; CBFB; SHOX2	Short Limb	[113]–[115]
MEF	Activating		MEF2c	Short Limb	[116]
HIF	Inhibitory		HIF1A	Short Limb	[117]
IRF	Inhibitory		IRF6	Short Limb	[118]–[120]
Chromatin Remodeling	Inhibitory		SATB2; JMJD3/KDM6B	Short Limb	[121], [122]
Chromatid Structure	Activating		DeltaEF1/ZEB1	Short Limb	[123]
	Inhibitory		NIPBL; SMC1a; HDAC8; RAD21; SMC3; PDS5B/APRIN	Short Limb	[124]–[134]
Extracellular Matrix					
Collagen	Inhibitory		COMP; Aggrecan; Col27a; JAWS; Col1a; PPIB; DDR2; CSF1; Mia3; TANGO1; Creb3L2/BBf2H7; Sec23a; Col2a	Short Limb	[4], [135]–[163]
Signaling	Inhibitory		Talpid (3); Ift88; Ift172	Short Limb	[164]–[166]
Sulfation	Inhibitory		PAPSS2; BPNT2; SMUF1; CHSY1; CSGALNACT1	Short Limb	[167]–[172]
Proteoglycans	Activating		SLC35D1; VCAN; HSPG2; Has2; FLNB; XYLT1; GUSMPS; GUS	Short Limb	[173]–[185]
MMP	Inhibitory		MT3-MMP; MT1-MMP; CDC42	Short Limb	[40], [186], [187]
Cell Physiology					
Cholesterol Synthesis	Inhibitory		Cyp26b1; SC5D; NSDHL	Short Limb	[188]–[192]
Lipid Formation	Inhibitory		DAPAT/DHAPAT/GNPAT	Short Limb	[193]–[199]
Bioelectricity	Inhibitory		TCIRG1; Clc7	Short Limb	[200]–[202]
Ca+ Signaling/Transport	Activating		TRPV4	Short Limb	[203], [204]
	Inhibitory		GP130; IFITM5/BRIL; TNNT3; Ano6/TMEM16F	Short Limb	[205]–[215]
Cell Cycle	Inhibitory		SFN	Short Limb	[216]
DNA Damage Repair	Inhibitory		Trp63/TP63	Short Limb	[217]

Note: While many of these mutations lead to multiple phenotypes, only the limb length phenotype is described in this table. References are in Supplementary File S1.

## Appendage Size Regulation During Limb Bud Development

### Transcription Factors

The alteration of a number of transcription factors have been found to impact limb length in mammals through their roles in patterning and differentiation of the limb bud. For example, Paired Related Homeobox 1 (Prx1 or Prrx1) is a homeobox transcription factor known for its role in mesodermal cell proliferation and fate in the developing limb. In an elegant experiment, the limb specific transcriptional enhancer of mouse *Prx1* was replaced by the orthologous enhancer from bat, *Carollia perspicillata* (Cretekos et al., 2008). This manipulation resulted in increased expression and an expansion of the expression domains of mouse *Prx1*, and an increase in the overall length of the mutant mouse limbs (Cretekos et al., 2008).

*HOX* genes, a group of highly conserved transcription factors that are essential for limb patterning also impact the length of the limb structures (Zakany and Duboule, 2007). Mouse knockouts of HoxD13, HoxA13, or HoxD12 result in both the truncation of the limb pattern and reduction of the overall limb size (Fromental-Ramain et al., 1996; Hérault et al., 1996; Cho et al., 2008). Increased and sustained expression of the *HoxD* locus occurs in the developing forelimb buds in bats. While these differences in expression do not result in noticeable differences in the growth and size of the fore and hind limb buds at the early stages, once differentiated, the skeletal elements in the autopod segment of bat forelimbs undergo a dramatic elongation, resulting in their proportionally larger size. Thus, loss of limb specific Hox genes appear to result in shortened limbs by negatively impacting pattern formation, while increased Hox expression positively correlates with limb size by increasing growth during the elongation stage of limb development.

Sox9 and paralogs Sox5 and Sox6 are members of the SRY-related HMG-box family of transcription factors and effect limb size through their regulation on chondrogenesis (Liu and Lefebvre, 2015). During embryonic limb development, Sox9 is considered the master chondrogenic factor, required for differentiation of mesenchymal precursor cells into chondrocytes (Lefebvre et al., 2001; Liu and Lefebvre, 2015). Sox9 then works in concert with Sox5 and Sox6 to drive differentiation and proliferation of chondrocytes (Lefebvre et al., 2001; Liu and Lefebvre, 2015). Activating mutations in Sox9 in mice results in a long limb phenotype (Long et al., 2020), while inhibiting mutations in the same gene results in short limb phenotypes (Akiyama et al., 2002, 2007). Furthermore, mouse knockouts of Sox5 and Sox6 in the limb bud mesenchyme results in chondrodysplasia with shortened limbs (Smits et al., 2001; Dy et al., 2008). These observations highlight the importance of this family of transcription factors on the regulation of growth during limb development.

### Genes Involved With Limb Skeletal Maturation

Once the limb bud is patterned and the skeletal tissues have differentiated, the regulation of the long bone growth plates greatly contributes to the overall size of the adult limb. The genes that regulate limb growth at this stage are involved with paracrine factor signaling. One example of this is Indian hedgehog (Ihh) signaling, which positively regulates cell proliferation within the growth plates of the long bones. When Ihh signaling is inactivated, through null *Ihh* or mutations in *Ihh* transducers or effectors, the resulting mammalian limbs are severely shortened (Mo et al., 1997; St-Jacques et al., 1999; Long et al., 2001; Razzaque et al., 2005; Ruiz-Perez et al., 2007; Sohaskey et al., 2008; Joeng and Long, 2009; Caparrós-Martín et al., 2013; Yoshida et al., 2015; Zhang et al., 2015; Kim et al., 2018). In contrast, overexpressing *Ihh* in the developing chick limb through viral transfection resulted in increased limb length (Bren-Mattison et al., 2011). These effects on limb size are generally tied to altered Ihh signaling during the processes of chondrocyte proliferation and differentiation and osteoblast differentiation in the growth plates in the long bones (Minina et al., 2002).

Interestingly, FGF activity has a differential impact on cell division depending on the stage of limb development. Studies in mammals and amphibians have shown that FGF signaling is essential for proliferation in the limb bud mesenchyme, while during post-embryonic limb maturation, FGFs participate in a negative feedback loop with Ihh in the growth plates (Coffin et al., 1995; Mancilla et al., 1998; Minina et al., 2002; Purushothaman et al., 2019). Gain-of-function mutations in both human and mice *FGFR3* result in achondroplasia characterized by a short limb phenotype (Iwata et al., 2000, 2001; Lee et al., 2017; Segev et al., 2000), while knockout of *FGFR3* in mice produces a long limb phenotype (Eswarakumar and Schlessinger, 2007; Toydemir et al., 2006; Tseng et al., 2010; Wen et al., 2016) (Table 1). Furthermore, knockout of *FGFR3* has been directly tied to increased Ihh and BMP signaling within the elongating skeletal tissue in mice (Wen et al., 2016).

BMPs also participate in a negative feedback loop with FGFs in the developing limb, and the inhibition of FGFs by BMPs is particularly important for the activation of *Sox9* expression, which is essential for chondrogenesis of the developing skeletal tissue in avian and mammalian limb buds (Chimal-Monroy et al., 2003; Yoon et al., 2006; Norrie et al., 2014). The negative feedback between BMP and FGF signaling is also present in the growth pates of mammalian long bones (Olsen et al., 2000; Chen et al., 2012; Studer et al., 2012; Wei et al., 2016). Overexpression of BMP2 and BMP4 ligands increases skeletal element size during chick limb development (Duprez et al., 1996). Moreover, inhibiting BMP signaling, via mutations in the receptors or downstream genes, leads to a shortened limb phenotype in mouse models (Evers et al., 1996; Barna et al., 2000; Settle et al., 2003; Klüppel et al., 2005; Lallemand et al., 2005; Aizawa et al., 2012; Bhattacharyya et al., 2015; Zhang et al., 2020). BMP signaling is essential for chondrocyte proliferation and differentiation in mouse growth plates (Yoon et al., 2006). Additionally, when BMP signaling is not present, *FGFR1* expression is elevated, which further represses the elongation of the long bones in both chicken and mouse models (Chimal-Monroy et al., 2003; Yoon et al., 2006; Norrie et al., 2014).

Both long and short limb phenotypes are additionally observed in mutations that affect TGFβ signaling. TGFβ’s regulate the construction and destruction of skeletal tissue by modulating the activity of osteoblasts and osteoclasts, respectively (Tang et al., 2009). Mutations in the human *TGFβ1* gene causes Camurati-Engelmann disease, one characteristic of which is elongated limbs (Kinoshita et al., 2000; Janssens et al., 2003). Additionally, mutations in *Fibrillin1* (*FBN1*), a TGFβ-binding partner, can lead to congenital syndromes (Marfan syndrome and Weill-Marchesani) in humans that result in either elongated or shortened limbs (Goff et al., 2011; Quarto et al., 2012). Fibrillin1 is an extracellular matrix glycoprotein necessary for microfibril associated signal transduction (Goff et al., 2011; Quarto et al., 2012). The human mutations largely reside in the TGFβ-binding domain, decreasing FBN1’s ability to sequester TGFβ ligands in the extracellular matrix, and increasing the bioavailability of TGFβ ligands (Goff et al., 2011; Quarto et al., 2012). It is unknown how the increased TGFβ activity observed in both Marfan syndrome and Weill-Marchesani syndromes lead to long and short limbs respectively, but the key difference might rely on the cell types that TGFβ signaling is hyperactivated in.

C-type natriuretic peptides, mostly known for their role in kidney function, also play a crucial role in limb sizing through chondrocyte regulation (Potter et al., 2009). Natriuretic peptide type C (NPC) activates the receptor (NPR-B or NPR2) to drive the synthesis of the second messenger, cGMP (Potter et al., 2009). In human patients, loss-of-function mutations in *NPR2* result in shortened limbs, while gain-of-function mutations cause Acromesomelic Dysplasia, Maroteaux Type, characterized by elongated limbs (Bartels et al., 2004; Faivre et al., 2000; Hannema et al., 2013; Ianakiev et al., 2000; Jiao et al., 2007; Kant et al., 1998; Lane and Dickie, 1968; Miura et al., 2012, 2014). The limb length phenotypes due to these mutations appear to be caused by effects on chondrocyte proliferation and differentiation, supporting the hypothesis that the regulation of the long bone growth pates is critical in determining overall scaling of the limb (Lane and Dickie, 1968; Kant et al., 1998; Faivre et al., 2000; Ianakiev et al., 2000; Bartels et al., 2004; Jiao et al., 2007; Potter et al., 2009; Miura et al., 2012, 2014; Hannema et al., 2013).

### Cell and Tissue Physiology Genes

Limb development and elongation requires that the cells are healthy enough to respond to the factors that regulate allometric growth. Thus, it is not surprising that gene mutations that negatively impact various aspects of cell physiology in the limb bud and immature limb will ultimately impact limb size. All the genes that fall under this category, including those that regulate lipid biosynthesis (Wanders et al., 1992; Clayton et al., 1994; Ofman et al., 1998; Thai et al., 2001; Rodemer et al., 2003; Nimmo et al., 2010; Itzkovitz et al., 2012), ion transport (Li et al., 1999; Kornak et al., 2001; Neutzsky-Wulff et al., 2008; Camacho et al., 2010; Weinstein et al., 2014), cell proliferation, and DNA damage repair (Vernersson Lindahl et al., 2013) have only been found to negatively impact limb size in mice and humans, suggesting that these factors may play permissive rather than instructive roles.

## Post-Embryonic Size Regulation

### Homeostasis

The maintenance of the appropriate limb size during tissue homeostasis depends on both the developmental stage of the animal, and whether it is a determinant or indeterminately growing species (Figure 1). In animals that have determinant growth, the lenth of limbs can be impacted up until the initiation of adulthood. In humans, limb elongation ends in late puberty, when the growth plates fuse and are no longer susceptible to the signals that promote their growth (reviewed in Shim, 2015). For example, altered nerve signaling in the limbs of pre-adult humans can result in a phenomenon known as macrodactyly, where one or more digits grows disproportionally larger than the other (Tsuge and Ikuta, 1973; Frykman and Wood, 1978; Razzaghi and Anastakis, 2005). In contrast, indeterminately growing species grow throughout their entire lives, and thus maintain active growth plates as adults (Riquelme-Guzmán et al., 2021). This indicates that growth plate activity must be continuously regulated in these limbs to maintain a size that is proportionally appropriate.

### Regeneration

Regeneration of adult limbs presents additional challenges that are nonexistent during embryonic/larval development. The injured limb is much larger than it was during embryonic development, and this larger size must be re-established to regain full function. While humans cannot regenerate their limbs, researchers are actively working to understand the mechanisms by which other species, such as the mouse and the Mexican axolotl (*Ambystoma mexicanum*), are capable of regenerating with the hopes that the knowledge is transferable to humans. While mice regenerate digit tips, the axolotl are able to regenerate complete limb structures (McCusker et al., 2015; Dolan et al., 2018). Thus, the factors that regulate the growth of the regenerate can have a large impact on the overall size of the limb in the axolotl model. Axolotl are also an indeterminately growing species. This creates an interesting paradigm since the regenerating limb must grow to a size larger than it was at the time of amputation to accommodate the animal’s growing body length. How this growth is regulated is unknown, and studies on this aspect of regeneration in the axolotl are challenging because of the extended period it takes for a regenerated limb to reach its “completed” size.

#### Blastema Development

Limb regeneration begins with the formation of a transient organ known as the limb blastema, which shares many molecular and functional similarities with the embryonic limb bud. Thus, it is reasonable to postulate that the modulation of factors that influence growth at this early stage in limb regeneration are conserved between limb development and regeneration. Because of the ease of loss of function approaches in the regenerating system, most of the manipulations that have led to sizing defects are a result of inhibition of signaling pathways that are essential during the early steps of blastema development. For example, pharmaceutical inhibition of FGF, BMP, or TGFβ signaling in the blastema all result in smaller limbs by impacting patterning, tissue differentiation, or the overall physiology in the blastema (Lévesque et al., 2007; Purushothaman et al., 2019; Vincent et al., 2020). Recently, it was observed that the repeated removal of the axolotl limb bud resulted in the formation of permanently miniaturized limbs (Bryant et al., 2017). Interestingly, these miniaturized limbs have a decreased abundance of limb nerves, which play a central role in the activation of key paracrine signals, such as FGFS and BMPs, during blastemal development (Makanae et al., 2014; Satoh et al., 2016; Bryant et al., 2017). Thus, the formation of the miniaturized size following limb amputation is likely related, in part, to diminished activation of these essential pathways.

To date, the only known signal that has been shown to positively influence the length of the regenerating limb is Retinoic Acid (RA). RA signaling is essential for pattern formation in both the embryonic and regenerating limb. Treatment of the regenerating limb with exogenous RA results in the elongation of the skeletal elements, and at high levels, causes the duplication of proximal/distal limb elements (Maden, 1983; Niazi et al., 1985). These phenotypes could be linked to the effect of RA on multiple transcription factors including HOXs that are essential in limb pattern formation (Gardiner and Bryant, 1996).

We have recently focused on the regulation of sizing of the axolotl limb regenerate during the maturation stages (Wells et al., 2021). Following the blastema stage of development, the regenerated limb is patterned and differentiated, yet is proportionally small. The regenerating limb then undergoes a phase of rapid growth until it reaches the size that is proportionally appropriate to the body size and is indistinguishable in length to the unamputated limb. Once the appropriate size is reached, the regenerated limb slows its rate of growth to match that of the rest of the animal (Wells et al., 2021). How the growth of the regenerating limb is regulated is only beginning to be elucidated, and our lab has recently discovered that signaling from the limb nerves play a key role in this process (Wells et al., 2021). Although the molecular mechanisms by which nerves control growth in the regenerate remain unknown, we speculate based on the above-described observations from developing limbs that they may impact the activity of the long bone growth plates. Additionally, one fascinating outstanding question is how the growth of the limb regenerate slows once the proportionally appropriate size has been reached.

## Summary

Tetrapods exhibit beautiful diversity in the proportionality, shape, and functionality of their limbs. Despite this, the underlying mechanisms that regulate limb growth, whether it is occurring in developing or regenerating limbs, appears to be well conserved. Mutant analyses indicate that factors that impact either limb bud patterning or the elongation of the long bones play the most important roles in limb size within an individual tetrapod species. So far, the limited data in regenerating limbs appears to follow the same rules, and thus studies in developing limbs can provide clues to better understanding post-embryonic limb growth. However, multiple aspects of regenerating limbs, such as how growth can be differentially regulated in a regenerating and non-injured limb on the same animal, and what the role of the nerves is in this regulation will likely only be resolved in regenerating species.
