# Supplementary material for: The Regulation of Growth in Developing, Homeostatic, and Regenerating Tetrapod Limbs: A Minireview
Source: Front Cell Dev Biol. 2022 Jan 3;9:768505. doi: 10.3389/fcell.2021.768505 (PMC8763381; doi:10.3389/fcell.2021.768505)
Supplement: Supplementary file 1 [file DataSheet1.doc]

[1] B. St-Jacques, M. Hammerschmidt, and A. P. McMahon, “Indian hedgehog signaling regulates proliferation and differentiation of chondrocytes and is essential for bone formation,” *Genes Dev.*, vol. 13, no. 16, 1999.

[2] M. S. Razzaque, D. W. Soegiarto, D. Chang, F. Long, and B. Lanske, “Conditional deletion of Indian hedgehog from collagen type 2α1-expressing cells results in abnormal endochondral bone formation,” *J. Pathol.*, vol. 207, no. 4, 2005.

[3] M. Yoshida *et al.*, “The transcription factor Foxc1 is necessary for Ihh-Gli2-regulated endochondral ossification,” *Nat. Commun.*, vol. 6, 2015.

[4] M. L. Sohaskey, J. Yu, M. A. Diaz, A. H. Plaas, and R. M. Harland, “JAWS coordinates chondrogenesis and synovial joint positioning,” *Development*, vol. 135, no. 13, 2008.

[5] F. Long, X. M. Zhang, S. Karp, Y. Yang, and A. P. McMahon, “Genetic manipulation of hedgehog signaling in the endochondral skeleton reveals a direct role in the regulation of chondrocyte proliferation,” *Development*, vol. 128, no. 24, 2001.

[6] V. L. Ruiz-Perez *et al.*, “Evc is a positive mediator of Ihh-regulated bone growth that localises at the base of chondrocyte cilia,” *Development*, vol. 134, no. 16, 2007.

[7] J. A. Caparrós-Martín *et al.*, “The ciliary EVC/EVC2 complex interacts with smo and controls hedgehog pathway activity in chondrocytes by regulating Sufu/Gli3 dissociation and Gli3 trafficking in primary cilia,” *Hum. Mol. Genet.*, vol. 22, no. 1, 2013.

[8] H. Zhang *et al.*, “Generation of Evc2/Limbin global and conditional KO mice and its roles during mineralized tissue formation,” *Genesis*, vol. 53, no. 9, 2015.

[9] Y. Kim *et al.*, “WDR11‐mediated Hedgehog signalling defects underlie a new ciliopathy related to Kallmann syndrome,” *EMBO Rep.*, vol. 19, no. 2, 2018.

[10] K. S. Joeng and F. Long, “The Gli2 transcriptional activator is a crucial effector for lhh signaling in osteoblast development and cartilage vascularization,” *Development*, vol. 136, no. 24, 2009.

[11] R. Mo *et al.*, “Specific and redundant functions of Gli2 and Gli3 zinc finger genes in skeletal patterning and development,” *Development*, vol. 124, no. 1, 1997.

[12] Y. Bren-Mattison, M. Hausburg, and B. B. Olwin, “Growth of limb muscle is dependent on skeletal-derived Indian hedgehog,” *Dev. Biol.*, vol. 356, no. 2, 2011.

[13] J. Böse, L. Grotewold, and U. Rüther, “Pallister-Hall syndrome phenotype in mice mutant for Gli3,” *Hum. Mol. Genet.*, vol. 11, no. 9, 2002.

[14] T. Cao, C. Wang, M. Yang, C. Wu, and B. Wang, “Mouse limbs expressing only the Gli3 repressor resemble those of Sonic hedgehog mutants,” *Dev. Biol.*, vol. 379, no. 2, 2013.

[15] S. A. Vokes, H. Ji, W. H. Wong, and A. P. McMahon, “A genome-scale analysis of the cis-regulatory circuitry underlying sonic hedgehog-mediated patterning of the mammalian limb,” *Genes Dev.*, vol. 22, no. 19, 2008.

[16] D. G. McFadden, J. McAnally, J. A. Richardson, J. Charité, and E. N. Olson, “Misexpression of dHAND induces ectopic digits in the developing limb bud in the absence of direct DNA binding,” *Development*, vol. 129, no. 13. 2002.

[17] D. C. Martinelli and C. Fan, “Gas1 extends the range of hedgehog action by facilitating its signaling,” *Dev. Biol.*, vol. 306, no. 1, 2007.

[18] H. Moon *et al.*, “Intestinal cell kinase, aprotein associated with endocrine-cerebro- osteodysplasia syndrome,is a key regulator of cilia length and Hedgehog signaling,” *Proc. Natl. Acad. Sci. U. S. A.*, vol. 111, no. 23, 2014.

[19] S. Yoshida *et al.*, “The novel ciliogenesis regulator dyrk2 governs hedgehog signaling during mouse embryogenesis,” *Elife*, vol. 9, 2020.

[20] P. B. Selby, S. N. Bolch, V. S. Mierzejewski, T. W. Mckinley, and G. D. Raymer, “Synergistic interactions between two skeletal mutations in mice: Individual and combined effects of the semidominants cleidocranial dysplasia (Ccd) and short digits (Dsh),” *J. Hered.*, vol. 84, no. 6, 1993.

[21] K. Handschuh *et al.*, “ESCRT-II/Vps25 constrains digit number by endosome-mediated selective modulation of FGF-SHH signaling,” *Cell Rep.*, vol. 9, no. 2, 2014.

[22] T. Iwata *et al.*, “A neonatal lethal mutation in FGFR3 uncouples proliferation and differentiation of growth plate chondrocytes in embryos,” *Hum. Mol. Genet.*, vol. 9, no. 11, 2000.

[23] T. Iwata, C. L. Li, C. X. Deng, and C. A. Francomano, “Highly activated Fgfr3 with the K644M mutation causes prolonged survival in severe dwarf mice,” *Hum. Mol. Genet.*, vol. 10, no. 12, 2001.

[24] Y. C. Lee, I. W. Song, Y. J. Pai, S. De Chen, and Y. T. Chen, “Knock-in human FGFR3 achondroplasia mutation as a mouse model for human skeletal dysplasia,” *Sci. Rep.*, vol. 7, 2017.

[25] O. Segev *et al.*, “Restrained chondrocyte proliferation and maturation with abnormal growth plate vascularization and ossification in human FGFR-3(G380R) transgenic mice,” *Hum. Mol. Genet.*, vol. 9, no. 2, 2000.

[26] V. P. Eswarakumar and J. Schlessinger, “Skeletal overgrowth is mediated by deficiency in a specific isoform of fibroblast growth factor receptor 3,” *Proc. Natl. Acad. Sci. U. S. A.*, vol. 104, no. 10, 2007.

[27] R. M. Toydemir *et al.*, “A novel mutation in FGFR3 causes camptodactyly, tall stature, and hearing loss (CATSHL) syndrome,” *Am. J. Hum. Genet.*, vol. 79, no. 5, 2006.

[28] M. Marinić, T. Aktas, S. Ruf, and F. Spitz, “An Integrated Holo-Enhancer Unit Defines Tissue and Gene Specificity of the Fgf8 Regulatory Landscape,” *Dev. Cell*, vol. 24, no. 5, 2013.

[29] D. Treichel, F. Schöck, H. Jäckle, P. Gruss, and A. Mansouri, “mBtd is required to maintain signaling during murine limb development,” *Genes Dev.*, vol. 17, no. 21, 2003.

[30] T. W. Bebee *et al.*, “The splicing regulators Esrp1 and Esrp2 direct an epithelial splicing program essential for mammalian development,” *Elife*, vol. 4, no. September2015, 2015.

[31] J. A. McMahon, S. Takada, L. B. Zimmerman, C. M. Fan, R. M. Harland, and A. P. McMahon, “Noggin-mediated antagonism of BMP signaling is required for growth and patterning of the neural tube and somite,” *Genes Dev.*, vol. 12, no. 10, 1998.

[32] J. A. Belo *et al.*, “Cerberus-like is a secreted BMP and nodal antagonist not essential for mouse development,” *Genesis*, vol. 26, no. 4, 2000.

[33] A. C. Borges, S. Marques, and J. A. Belo, “The BMP antagonists cerberus-like and noggin do not interact during mouse forebrain development,” *Int. J. Dev. Biol.*, vol. 45, no. 2, 2001.

[34] D. Duprez *et al.*, “Overexpression of BMP-2 and BMP-4 alters the size and shape of developing skeletal elements in the chick limb,” *Mech. Dev.*, vol. 57, no. 2, 1996.

[35] H. Zhang *et al.*, “Loss of BMP signaling mediated by BMPR1A in osteoblasts leads to differential bone phenotypes in mice depending on anatomical location of the bones,” *Bone*, vol. 137, 2020.

[36] S. H. Settle, R. B. Rountree, A. Sinha, A. Thacker, K. Higgins, and D. M. Kingsley, “Multiple joint and skeletal patterning defects caused by single and double mutations in the mouse Gdf6 and Gdf5 genes,” *Dev. Biol.*, vol. 254, no. 1, 2003.

[37] M. Evers *et al.*, “Targeted disruption of the arylsulfatase B gene results in mice resembling the phenotype of mucopolysaccharidosis VI,” *Proc. Natl. Acad. Sci. U. S. A.*, vol. 93, no. 16, 1996.

[38] S. Bhattacharyya, L. Feferman, and J. K. Tobacman, “Regulation of chondroitin-4-sulfotransferase (CHST11) expression by opposing effects of arylsulfatase B on BMP4 and Wnt9A,” *Biochim. Biophys. Acta - Gene Regul. Mech.*, vol. 1849, no. 3, 2015.

[39] Y. Lallemand, M. A. Nicola, C. Ramos, A. Bach, C. Saint Cloment, and B. Robert, “Analysis of Msx1; Msx2 double mutants reveals multiple roles for Msx genes in limb development,” *Development*, vol. 132, no. 13, 2005.

[40] R. Aizawa *et al.*, “Cdc42 is required for chondrogenesis and interdigital programmed cell death during limb development,” *Mech. Dev.*, vol. 129, no. 1–4, 2012.

[41] M. Barna, N. Hawe, N. Lee, and P. P. Pandolfi, “Plzf regulates limb and axial skeletal patterning,” *Nat. Genet.*, vol. 25, no. 2, 2000.

[42] M. Klüppel, T. N. Wight, C. Chan, A. Hinek, and J. L. Wrana, “Maintenance of chondroitin sulfation balance by chondroitin-4-sulfotransferase 1 is required for chondrocyte development and growth factor signaling during cartilage morphogenesis,” *Development*, vol. 132, no. 17, 2005.

[43] A. J. Doyle *et al.*, “Mutations in the TGF-β repressor SKI cause Shprintzen-Goldberg syndrome with aortic aneurysm,” *Nat. Genet.*, vol. 44, no. 11, 2012.

[44] K. Janssens, P. Ten Dijke, S. H. Ralston, C. Bergmann, and W. Van Hul, “Transforming growth factor-β1 mutations in Camurati-Engelmann disease lead to increased signaling by altering either activation or secretion of the mutant protein,” *J. Biol. Chem.*, vol. 278, no. 9, 2003.

[45] A. Kinoshita *et al.*, “Domain-specific mutations in TGFB1 result in Camurati-Engelmann disease,” *Nat. Genet.*, vol. 26, no. 1, 2000.

[46] C. Le Goff *et al.*, “Mutations in the TGFβb binding-protein-like domain 5 of FBN1 are responsible for acromicric and geleophysic dysplasias,” *Am. J. Hum. Genet.*, vol. 89, no. 1, 2011.

[47] N. Quarto, S. Li, A. Renda, and M. T. Longaker, “Exogenous activation of BMP-2 signaling overcomes TGFβ-mediated inhibition of osteogenesis in marfan embryonic stem cells and marfan patient-specific induced pluripotent stem cells,” *Stem Cells*, vol. 30, no. 12, 2012.

[48] S. E. Hannema *et al.*, “An activating mutation in the kinase homology domain of the natriuretic peptide receptor-2 causes extremely tall stature without skeletal deformities,” *J. Clin. Endocrinol. Metab.*, vol. 98, no. 12, 2013.

[49] K. Miura *et al.*, “An overgrowth disorder associated with excessive production of cgmp due to a gain-of-function mutation of the natriuretic peptide receptor 2 gene,” *PLoS One*, vol. 7, no. 8, 2012.

[50] K. Miura *et al.*, “Overgrowth syndrome associated with a gain-of-function mutation of the natriuretic peptide receptor 2 (NPR2) gene,” *Am. J. Med. Genet. Part A*, vol. 164, no. 1, 2014.

[51] C. F. Bartels *et al.*, “Mutations in the transmembrane natriuretic peptide receptor NPR-B impair skeletal growth and cause acromesomelic dysplasia, type Maroteaux,” *Am. J. Hum. Genet.*, vol. 75, no. 1, 2004.

[52] L. Faivre *et al.*, “Exclusion of chromosome 9 helps to identify mild variants of acromesomelic dysplasia Maroteaux type,” *Journal of Medical Genetics*, vol. 37, no. 1. 2000.

[53] P. Ianakiev *et al.*, “Localization of an acromesomelic dysplasia on chromosome 9 by homozygosity mapping,” *Clin. Genet.*, vol. 57, no. 4, 2000.

[54] Y. Jiao *et al.*, “A single nucleotide mutation in Nppc is associated with a long bone abnormality in lbab mice,” *BMC Genet.*, vol. 8, 2007.

[55] S. G. Kant *et al.*, “Acromesomelic dysplasia maroteaux type maps to human chromosome 9,” *Am. J. Hum. Genet.*, vol. 63, no. 1, 1998.

[56] P. W. Lane and M. M. Dickie, “Three recessive mutations producing disproportionate dwarfing in mice: Aehondroplasia, brachymorphic, and stubby,” *J. Hered.*, vol. 59, no. 5, 1968.

[57] K. Takeda *et al.*, “Limb and skin abnormalities in mice lacking IKKalpha [see comments],” *Science (80-. ).*, vol. 284, no. 5412, 1999.

[58] S. Yang and Y. P. Li, “RGS10-null mutation impairs osteoclast differentiation resulting from the loss of [Ca2+]i oscillation regulation,” *Genes Dev.*, vol. 21, no. 14, 2007.

[59] P. M. Holland *et al.*, “RIP4 is an ankyrin repeat-containing kinase essential for keratinocyte differentiation,” *Curr. Biol.*, vol. 12, no. 16, 2002.

[60] W. C. Dougall *et al.*, “RANK is essential for osteoclast and lymph node development,” *Genes Dev.*, vol. 13, no. 18, 1999.

[61] A. Leibbrandt and J. M. Penninger, “RANK/RANKL: Regulators of immune responses and bone physiology,” *Annals of the New York Academy of Sciences*, vol. 1143. 2008.

[62] W. Satoh, T. Gotoh, Y. Tsunematsu, S. Aizawa, and A. Shimon, “Sfrp 1 and Sfrp2 regulate anteroposterior axis elongation and somite segmentation during mouse embryogenesis,” *Development*, vol. 133, no. 6, 2006.

[63] H. H. Lee and R. R. Behringer, “Conditional expression of Wnt4 during chondrogenesis leads to dwarfism in mice,” *PLoS One*, vol. 2, no. 5, 2007.

[64] T. P. Yamaguchi, A. Bradley, A. P. McMahon, and S. Jones, “A Wnt5a pathway underlies outgrowth of multiple structures in the vertebrate embryo,” *Development*, vol. 126, no. 6, 1999.

[65] C. J. Zhou, Y. Z. Wang, T. Yamagami, T. Zhao, L. Song, and K. Wang, “Generation of Lrp6 conditional gene-targeting mouse line for modeling and dissecting multiple birth defects/congenital anomalies,” *Dev. Dyn.*, vol. 239, no. 1, 2010.

[66] T. M. DeChiara *et al.*, “Ror2, encoding a receptor-like tyrosine kinase, is required for cartilage and growth plate development,” *Nat. Genet.*, vol. 24, no. 3, 2000.

[67] S. Wu, G. Ying, Q. Wu, and M. R. Capecchi, “Toward simpler and faster genome-wide mutagenesis in mice,” *Nat. Genet.*, vol. 39, no. 7, 2007.

[68] C. Liu, C. Lin, C. Gao, H. May-Simera, A. Swaroop, and T. Li, “Null and hypomorph Prickle1 alleles in mice phenocopy human Robinow syndrome and disrupt signaling downstream of Wnt5a,” *Biol. Open*, vol. 3, no. 9, 2014.

[69] S. Neufeld *et al.*, “A conditional allele of Rspo3 reveals redundant function of R-spondins during mouse limb development,” *Genesis*, vol. 50, no. 10. 2012.

[70] M. M. Halford *et al.*, “Ryk-deficient mice exhibit craniofacial defects associated with perturbed Eph receptor crosstalk,” *Nat. Genet.*, vol. 25, no. 4, 2000.

[71] X. Zhu *et al.*, “Wls-mediated Wnts differentially regulate distal limb patterning and tissue morphogenesis,” *Dev. Biol.*, vol. 365, no. 2, 2012.

[72] S. J. Rodda and A. P. McMahon, “Distinct roles for Hedgehog and caronical Wnt signaling in specification, differentiation and maintenance of osteoblast progenitors,” *Development*, vol. 133, no. 16, 2006.

[73] J. J. Barrott, G. M. Cash, A. P. Smith, J. R. Barrow, and L. C. Murtaugh, “Deletion of mouse Porcn blocks Wnt ligand secretion and reveals an ectodermal etiology of human focal dermal hypoplasia/Goltz syndrome,” *Proc. Natl. Acad. Sci. U. S. A.*, vol. 108, no. 31, 2011.

[74] W. Liu *et al.*, “Deletion of porcn in mice leads to multiple developmental defects and models human focal dermal hypoplasia (Goltz syndrome),” *PLoS One*, vol. 7, no. 3, 2012.

[75] D. Bornholdt *et al.*, “PORCN mutations in focal dermal hypoplasia: Coping with lethality,” *Hum. Mutat.*, vol. 30, no. 5, 2009.

[76] K. H. Grzeschik *et al.*, “Deficiency of PORCN, a regulator of Wnt signaling, is associated with focal dermal hypoplasia,” *Nat. Genet.*, vol. 39, no. 7, 2007.

[77] P. Leoyklang, K. Suphapeetiporn, S. Wananukul, and V. Shotelersuk, “Three novel mutations in the PORCN gene underlying focal dermal hypoplasia,” *Clin. Genet.*, vol. 73, no. 4, 2008.

[78] X. Wang *et al.*, “Mutations in X-linked PORCN, a putative regulator of Wnt signaling, cause focal dermal hypoplasia,” *Nat. Genet.*, vol. 39, no. 7, 2007.

[79] H. Y. H. Ho *et al.*, “Wnt5a-Ror-Dishevelled signaling constitutes a core developmental pathway that controls tissue morphogenesis,” *Proc. Natl. Acad. Sci. U. S. A.*, vol. 109, no. 11, 2012.

[80] E. C. Weir, W. M. Philbrick, M. Amling, L. A. Neff, R. Baron, and A. E. Broadus, “Targeted overexpression of parathyroid hormone-related peptide in chondrocytes causes chondrodysplasia and delayed endochondral bone formation,” *Proc. Natl. Acad. Sci. U. S. A.*, vol. 93, no. 19, 1996.

[81] F. Csukasi *et al.*, “The PTH/PTHrP-SIK3 pathway affects skeletogenesis through altered mTOR signaling,” *Sci. Transl. Med.*, vol. 10, no. 459, 2018.

[82] E. Schipani, K. Kruse, and H. Jüppner, “A constitutively active mutant PTH-PTHrP receptor in Jansen-type metaphyseal chondrodysplasia,” *Science (80-. ).*, vol. 268, no. 5207, 1995.

[83] E. Schipani *et al.*, “Targeted expression of constitutively active receptors for parathyroid hormone and parathyroid hormone-related peptide delays endochondral bone formation and rescues mice that lack parathyroid hormone-related peptide,” *Proc. Natl. Acad. Sci. U. S. A.*, vol. 94, no. 25, 1997.

[84] A. C. Karaplis *et al.*, “Lethal skeletal dysplasia from targeted disruption of the parathyroid hormone-related peptide gene,” *Genes Dev.*, vol. 8, no. 3, 1994.

[85] B. Lanske *et al.*, “PTH/PTHrP receptor in early development and Indian hedgehog-regulated bone growth,” *Science*, vol. 273, no. 5275. 1996.

[86] N. Amizuka *et al.*, “Signalling by fibroblast growth factor receptor 3 and parathyroid hormone-related peptide coordinate cartilage and bone development,” *Bone*, vol. 34, no. 1, 2004.

[87] D. Miao, B. He, A. C. Karaplis, and D. Goltzman, “Parathyroid hormone is essential for normal fetal bone formation,” *J. Clin. Invest.*, vol. 109, no. 9, 2002.

[88] W. M. Philbrick, B. E. Dreyer, I. A. Nakchbandi, and A. C. Karaplis, “Parathyroid hormone-related protein is required for tooth eruption,” *Proc. Natl. Acad. Sci. U. S. A.*, vol. 95, no. 20, 1998.

[89] A. Sakamoto, M. Chen, T. Kobayashi, H. M. Kronenberg, and L. S. Weinstein, “Chondrocyte-specific knockout of the G protein Gsα leads to epiphyseal and growth plate abnormalities and ectopic chondrocyte formation,” *J. Bone Miner. Res.*, vol. 20, no. 4, 2005.

[90] A. S. Jobert *et al.*, “Absence of functional receptors for parathyroid hormone and parathyroid hormone-related peptide in Blomstrand chondrodysplasia,” *J. Clin. Invest.*, vol. 102, no. 1, 1998.

[91] J. J. Wysolmerski *et al.*, “Absence of functional type 1 parathyroid hormone (PTH)/PTH-related protein receptors in humans is associated with abnormal breast development and tooth impaction,” *J. Clin. Endocrinol. Metab.*, vol. 86, no. 4, 2001.

[92] J. H. D. Bassett *et al.*, “A lack of thyroid hormones rather than excess thyrotropin causes abnormal skeletal development in hypothyroidism,” *Mol. Endocrinol.*, vol. 22, no. 2, 2008.

[93] I. M. Bird *et al.*, “The skeletal phenotype of achondrogenesis type 1A is caused exclusively by cartilage defects,” *Dev.*, vol. 145, no. 1, 2018.

[94] J. Bonaventure *et al.*, “Type II collagen defect in two sibs with the Goldblatt syndrome, a chondrodysplasia with dentinogenesis imperfecta, and joint laxity,” *Am. J. Med. Genet.*, vol. 44, no. 6, 1992.

[95] C. T. N. Medina, R. Sandoval, G. Oliveira, K. da Costa Silveira, D. P. Cavalcanti, and R. Pogue, “Pathogenic variants in the TRIP11 gene cause a skeletal dysplasia spectrum from odontochondrodysplasia to achondrogenesis 1A,” *Am. J. Med. Genet. Part A*, vol. 182, no. 4, 2020.

[96] A. Wehrle *et al.*, “Hypomorphic mutations of TRIP11 cause odontochondrodysplasia,” *JCI Insight*, vol. 4, no. 3, 2019.

[97] P. Smits *et al.*, “Lethal Skeletal Dysplasia in Mice and Humans Lacking the Golgin GMAP-210,” *N. Engl. J. Med.*, vol. 362, no. 3, 2010.

[98] S. Vanegas, L. F. Sua, J. López-Tenorio, D. Ramírez-Montaño, and H. Pachajoa, “Achondrogenesis type 1A: Clinical, histologic, molecular, and prenatal ultrasound diagnosis,” *Appl. Clin. Genet.*, vol. 11, 2018.

[99] J. H. D. Bassett *et al.*, “Thyroid hormone excess rather than thyrotropin deficiency induces osteoporosis in hyperthyroidism,” *Mol. Endocrinol.*, vol. 21, no. 5, 2007.

[100] C. J. Cretekos, Y. Wang, E. D. Green, J. F. Martin, J. J. Rasweiler IV, and R. R. Behringer, “Regulatory divergence modifies limb length between mammals,” *Genes Dev.*, vol. 22, no. 2, 2008.

[101] K. W. Cho, J. Y. Kim, J. W. Cho, K. H. Cho, C. W. Song, and H. S. Jung, “Point mutation of Hoxd12 in mice,” *Yonsei Med. J.*, vol. 49, no. 6, 2008.

[102] C. Fromental-Ramain, X. Warot, N. Messadecq, M. LeMeur, P. Dollé, and P. Chambon, “Hoxa-13 and Hoxd-13 play a crucial role in the patterning of the limb autopod,” *Development*, vol. 122, no. 10, 1996.

[103] Y. Hérault, S. Hraba-Renevey, F. Van Der Hoeven, and D. Duboule, “Function of the Evx-2 gene in the morphogenesis of vertebrate limbs,” *EMBO J.*, vol. 15, no. 23, 1996.

[104] H. Song *et al.*, “Mammalian Mst1 and Mst2 kinases play essential roles in organ size control and tumor suppression,” *Proc. Natl. Acad. Sci. U. S. A.*, vol. 107, no. 4, 2010.

[105] H. K. Long *et al.*, “Loss of Extreme Long-Range Enhancers in Human Neural Crest Drives a Craniofacial Disorder,” *Cell Stem Cell*, vol. 27, no. 5, 2020.

[106] P. Dy, Y. Han, and V. Lefebvre, “Generation of mice harboring a Sox5 conditional null allele,” *Genesis*, vol. 46, no. 6, 2008.

[107] P. Smits *et al.*, “The Transcription Factors L-Sox5 and Sox6 Are Essential for Cartilage Formation,” *Dev. Cell*, vol. 1, no. 2, 2001.

[108] H. Akiyama, M. C. Chaboissier, J. F. Martin, A. Schedl, and B. De Crombrugghe, “The transcription factor Sox9 has essential roles in successive steps of the chondrocyte differentiation pathway and is required for expression of Sox5 and Sox6,” *Genes Dev.*, vol. 16, no. 21, 2002.

[109] H. Akiyama *et al.*, “Misexpression of Sox9 in mouse limb bud mesenchyme induces polydactyly and rescues hypodactyly mice,” *Matrix Biol.*, vol. 26, no. 4, 2007.

[110] C. Wu *et al.*, “Kindlin-2 controls TGF-β signalling and Sox9 expression to regulate chondrogenesis,” *Nat. Commun.*, vol. 6, 2015.

[111] P. Bialek *et al.*, “A twist code determines the onset of osteoblast differentiation,” *Dev. Cell*, vol. 6, no. 3, 2004.

[112] R. M. Guzzo, V. Andreeva, D. B. Spicer, and M. H. Drissi, “Persistent expression of Twist1 in chondrocytes causes growth plate abnormalities and dwarfism in mice,” *Int. J. Dev. Biol.*, vol. 55, no. 6, 2011.

[113] J. Cobb, A. Dierich, Y. Huss-Garcia, and D. Duboule, “A mouse model for human short-stature syndromes identifies Shox2 as an upstream regulator of Runx2 during long-bone development,” *Proc. Natl. Acad. Sci. U. S. A.*, vol. 103, no. 12, 2006.

[114] T. Komori *et al.*, “Targeted disruption of Cbfa1 results in a complete lack of bone formation owing to maturational arrest of osteoblasts,” *Cell*, vol. 89, no. 5, 1997.

[115] J. Miller *et al.*, “The core-binding factor β subunit is required for bone formation and hematopoietic maturation,” *Nat. Genet.*, vol. 32, no. 4, 2002.

[116] M. A. Arnold *et al.*, “MEF2C Transcription Factor Controls Chondrocyte Hypertrophy and Bone Development,” *Dev. Cell*, vol. 12, no. 3, 2007.

[117] E. Schipani, H. E. Ryan, S. Didrickson, T. Kobayashi, M. Knight, and R. S. Johnson, “Hypoxia in cartilage: HIF-1α is essential for chondrocyte growth arrest and survival,” *Genes Dev.*, vol. 15, no. 21, 2001.

[118] C. R. Ingraham *et al.*, “Abnormal skin, limb and craniofacial morphogenesis in mice deficient for interferon regulatory factor 6 (Irf6),” *Nat. Genet.*, vol. 38, no. 11, 2006.

[119] R. J. Richardson *et al.*, “Irf6 is a key determinant of the keratinocyte proliferation-differentiation switch,” *Nat. Genet.*, vol. 38, no. 11, 2006.

[120] R. W. Stottmann, B. C. Bjork, J. B. Doyle, and D. R. Beier, “Identification of a Van Der Woude Syndrome mutation in the Cleft Palate 1 mutant mouse,” *Genesis*, vol. 48, no. 5, 2010.

[121] O. Britanova *et al.*, “Satb2 haploinsufficiency phenocopies 2q32-q33 deletions, whereas loss suggests a fundamental role in the coordination of jaw development,” *Am. J. Hum. Genet.*, vol. 79, no. 4, 2006.

[122] F. Zhang *et al.*, “JMJD3 promotes chondrocyte proliferation and hypertrophy during endochondral bone formation in mice,” *J. Mol. Cell Biol.*, vol. 7, no. 1, 2015.

[123] T. Takagi, H. Moribe, H. Kondoh, and Y. Higashi, “δEF1, a zinc finger and homeodomain transcription factor, is required for skeleton patterning in multiple lineages,” *Development*, vol. 125, no. 1, 1998.

[124] Z. A. Bhuiyan *et al.*, “Genotype-phenotype correlations of 39 patients with Cornelia de Lange syndrome: The Dutch experience,” *J. Med. Genet.*, vol. 43, no. 7, 2006.

[125] M. Ireland and J. Burn, “Cornelia de Lange syndrome - Photo essay,” *Clin. Dysmorphol.*, vol. 2, no. 2, 1993.

[126] B. Zhang *et al.*, “Mice lacking sister chromatid cohesion protein PDS5B exhibit developmental abnormalities reminiscent of Cornelia de Lange syndrome,” *Development*, vol. 134, no. 17, 2007.

[127] I. D. Krantz *et al.*, “Exclusion of linkage to the CDL1 gene region on chromosome 3q26.3 in some familial cases of Cornelia de Lange Syndrome,” *Am. J. Med. Genet.*, vol. 101, no. 2, 2001.

[128] I. D. Krantz *et al.*, “Cornelia de Lange syndrome is caused by mutations in NIPBL, the human homolog of Drosophila melanogaster Nipped-B,” *Nat. Genet.*, vol. 36, no. 6, 2004.

[129] D. Pehlivan *et al.*, “NIPBL rearrangements in Cornelia de Lange syndrome: Evidence for replicative mechanism and genotype-phenotype correlation,” *Genet. Med.*, vol. 14, no. 3, 2012.

[130] J. Pié *et al.*, “Mutations and variants in the cohesion factor genes NIPBL, SMC1A, and SMC3 in a cohort of 30 unrelated patients with Cornelia de Lange syndrome,” *Am. J. Med. Genet. Part A*, vol. 152, no. 4, 2010.

[131] A. Selicorni *et al.*, “Clinical score of 62 Italian patients with Cornelia de Lange syndrome and correlations with the presence and type of NIPBL mutation,” *Clin. Genet.*, vol. 72, no. 2, 2007.

[132] E. T. Tonkin, T. J. Wang, S. Lisgo, M. J. Bamshad, and T. Strachan, “NIPBL, encoding a homolog of fungal Scc2-type sister chromatid cohesion proteins and fly Nipped-B, is mutated in Cornelia de Lange syndrome,” *Nat. Genet.*, vol. 36, no. 6, 2004.

[133] E. T. Tonkin *et al.*, “A giant novel gene undergoing extensive alternative splicing is severed by a Cornelia de Lange-associated translocation breakpoint at 3q26.3,” *Hum. Genet.*, vol. 115, no. 2, 2004.

[134] W. G. Wilson, J. M. Kennaugh, J. P. Kugler, and H. E. Wyandt, “Reciprocal translocation 14q;21q in a patient with the Brachmann-de Lange syndrome,” *J. Med. Genet.*, vol. 20, no. 6, 1983.

[135] M. A. Flynn and R. M. Pauli, “Double heterozygosity in bone growth disorders: Four new observations and review,” *American Journal of Medical Genetics*, vol. 121 A, no. 3. 2003.

[136] R. T. Cowling *et al.*, “Discoidin domain receptor 2 germline gene deletion leads to altered heart structure and function in the mouse,” *Am. J. Physiol. - Hear. Circ. Physiol.*, vol. 307, no. 5, 2014.

[137] X. M. Dai *et al.*, “Targeted disruption of the mouse colony-stimulating factor 1 receptor gene results in osteopetrosis, mononuclear phagocyte deficiency, increased primitive progenitor cell frequencies, and reproductive defects,” *Blood*, vol. 99, no. 1, 2002.

[138] D. G. Wilson *et al.*, “Global defects in collagen secretion in a Mia3/TANGO1 knockout mouse,” *J. Cell Biol.*, vol. 193, no. 5, 2011.

[139] K. G. Gaiser *et al.*, “Y-position collagen II mutation disrupts cartilage formation and skeletal development in a transgenic mouse model of spondyloepiphyseal dysplasia,” *J. Bone Miner. Res.*, vol. 17, no. 1, 2002.

[140] A. Saito *et al.*, “Regulation of endoplasmic reticulum stress response by a BBF2H7-mediated Sec23a pathway is essential for chondrogenesis,” *Nat. Cell Biol.*, vol. 11, no. 10, 2009.

[141] R. Dahiya, S. Cleveland, and C. A. Megerian, “Spondyloepiphyseal dysplasia congenita associated with conductive hearing loss,” *Ear, Nose Throat J.*, vol. 79, no. 3, 2000.

[142] K. C. Silveira, L. C. Bonadia, A. Superti-Furga, D. R. Bertola, A. A. L. Jorge, and D. P. Cavalcanti, “Six additional cases of SEDC due to the same and recurrent R989C mutation in the COL2A1 gene-the clinical and radiological follow-up,” *Am. J. Med. Genet. Part A*, vol. 167, no. 4, 2015.

[143] J. Spranger, A. Winterpacht, and B. Zabel, “The type II collagenopathies: A spectrum of chondrodysplasias,” *European Journal of Pediatrics*, vol. 153, no. 2. 1994.

[144] P. A. Terhal *et al.*, “Mutation-based growth charts for SEDC and other COL2A1 related dysplasias,” *Am. J. Med. Genet. Part C Semin. Med. Genet.*, vol. 160 C, no. 3, 2012.

[145] X. Xia *et al.*, “A first familial G504S mutation of COL2A1 gene results in distinctive spondyloepiphyseal dysplasia congenita,” *Clinica Chimica Acta*, vol. 382, no. 1–2. 2007.

[146] L. O. Langer, G. B. Schaefer, and D. T. Wadsworth, “Patient with double heterozygosity for achondroplasia and pseudoachondroplasia, with comments on these conditions and the relationship between pseudoachondroplasia and multiple epiphyseal dysplasia, Fairbank type,” *Am. J. Med. Genet.*, vol. 47, no. 5, 1993.

[147] Z. Zhang, J. W. He, W. Z. Fu, C. Q. Zhang, and Z. L. Zhang, “Identification of three novel mutations in the COL2A1 gene in four unrelated Chinese families with spondyloepiphyseal dysplasia congenita,” *Biochem. Biophys. Res. Commun.*, vol. 413, no. 4, 2011.

[148] R. Ballo, P. H. Beighton, and R. S. Ramesar, “Stickler-like syndrome due to a dominant negative mutation in the COL2A1 gene,” *Am. J. Med. Genet.*, vol. 80, no. 1, 1998.

[149] P. Beighton, L. Goldberg, and J. O. t. Hof, “Dominant inheritance of multiple epiphyseal dysplasia, myopia and deafness,” *Clin. Genet.*, vol. 14, no. 3, 1978.

[150] G. Liang *et al.*, “Endoplasmic reticulum stress-unfolding protein response-apoptosis cascade causes chondrodysplasia in a col2a1 p.Gly1170Ser mutated mouse model,” *PLoS One*, vol. 9, no. 1, 2014.

[151] G. R. Mortler *et al.*, “A radiographic, morphologic, biochemical and molecular analysis of a case of achondrogenesis type II resulting from substitution for a glycine residue (Gly691→Arg) in the type II collagen trimer,” *Hum. Mol. Genet.*, vol. 4, no. 2, 1995.

[152] J. Spranger, A. Winterpacht, and B. Zabel, “Kniest dysplasia: Dr. W. Kniest, his patient, the molecular defect,” *Am. J. Med. Genet.*, vol. 69, no. 1, 1997.

[153] D. J. Wilkin, R. Bogaert, R. S. Lachman, D. L.rimoin, D. R. Eyre, and D. H. Cohn, “A single amino acid substitution (G103D) in the type II collagen triple helix produces kniest dysplasia,” *Hum. Mol. Genet.*, vol. 3, no. 11, 1994.

[154] A. Winterpacht, M. Hilbert, U. Schwarze, S. Mundlos, J. Spranger, and B. U. Zabel, “Kniest and Stickler dysplasia phenotypes caused by collagen type II gene (COL2A1) defect,” *Nat. Genet.*, vol. 3, no. 4, 1993.

[155] K. S. Brown, R. E. Cranley, and R. Greene, “Disproportionate micromelia (Dmm): An incomplete dominant mouse dwarfism with abnormal cartilage matrix,” *J. Embryol. Exp. Morphol.*, vol. Vol. 62, 1981.

[156] M. Cionni, C. Menke, and R. W. Stottmann, “The mouse MC13 mutant is a novel ENU mutation in collagen type II, alpha 1,” *PLoS One*, vol. 9, no. 12, 2014.

[157] C. G. Woods, J. G. Rogers, and V. Mayne, “Two sibs who are double heterozygotes for achondroplasia and pseudoachondroplastic dysplasia,” *J. Med. Genet.*, vol. 31, no. 7, 1994.

[158] K. L. Lauing, M. Cortes, M. S. Domowicz, J. G. Henry, A. T. Baria, and N. B. Schwartz, “Aggrecan is required for growth plate cytoarchitecture and differentiation,” *Dev. Biol.*, vol. 396, no. 2, 2014.

[159] K. A. Piróg-Garcia *et al.*, “Reduced cell proliferation and increased apoptosis are significant pathological mechanisms in a murine model of mild pseudoachondroplasia resulting from a mutation in the C-terminal domain of COMP,” *Hum. Mol. Genet.*, vol. 16, no. 17, 2007.

[160] S. Unger, J. Korkko, D. Krakow, R. S. Lachman, D. L. Rimoin, and D. H. Cohn, “Double heterozygosity for pseudoachondroplasia and spondyloepiphyseal dysplasia congenita,” *Am. J. Med. Genet.*, vol. 104, no. 2, 2001.

[161] D. A. Plumb *et al.*, “Collagen XXVII organises the pericellular matrix in the growth plate,” *PLoS One*, vol. 6, no. 12, 2011.

[162] K. Tabeta *et al.*, “An ENU-induced splice site mutation of mouse Col1a1 causing recessive osteogenesis imperfecta and revealing a novel splicing rescue,” *Sci. Rep.*, vol. 7, no. 1, 2017.

[163] W. A. Cabral *et al.*, “Abnormal Type I Collagen Post-translational Modification and Crosslinking in a Cyclophilin B KO Mouse Model of Recessive Osteogenesis Imperfecta,” *PLoS Genet.*, vol. 10, no. 6, 2014.

[164] F. Bangs *et al.*, “Generation of mice with functional inactivation of talpid3, a gene first identified in chicken,” *Development*, vol. 138, no. 15, 2011.

[165] C. J. Haycraft *et al.*, “Intraflagellar transport is essential for endochondral bone formation,” *Development*, vol. 134, no. 2, 2007.

[166] P. W. Howard, T. L. Howard, and R. A. Maurer, “Generation of mice with a conditional allele for Ift172,” *Transgenic Res.*, vol. 19, no. 1, 2010.

[167] M. Cortes, A. T. Baria, and N. B. Schwartz, “Sulfation of chondroitin sulfate proteoglycans is necessary for proper Indian hedgehog signaling in the developing growth plate,” *Development*, vol. 136, no. 10, 2009.

[168] J. P. Frederick *et al.*, “A role for a lithium-inhibited Golgi nucleotidase in skeletal development and sulfation,” *Proc. Natl. Acad. Sci. U. S. A.*, vol. 105, no. 33, 2008.

[169] C. Settembre *et al.*, “Systemic inflammation and neurodegeneration in a mouse model of multiple sulfatase deficiency,” *Proc. Natl. Acad. Sci. U. S. A.*, vol. 104, no. 11, 2007.

[170] D. G. Wilson *et al.*, “Chondroitin sulfate synthase 1 (Chsy1) is required for bone development and digit patterning,” *Dev. Biol.*, vol. 363, no. 2, 2012.

[171] T. Sato *et al.*, “Chondroitin sulfate N-acetylgalactosaminyltransferase 1 is necessary for normal endochondral ossification and aggrecan metabolism,” *J. Biol. Chem.*, vol. 286, no. 7, 2011.

[172] Y. Watanabe *et al.*, “Chondroitin sulfate N-acetylgalactosaminyltransferase-1 is required for normal cartilage development,” *Biochem. J.*, vol. 432, no. 1, 2010.

[173] K. Choocheep *et al.*, “Versican facilitates chondrocyte differentiation and regulates joint morphogenesis,” *J. Biol. Chem.*, vol. 285, no. 27, 2010.

[174] S. Hiraoka *et al.*, “Nucleotide-sugar transporter SLC35D1 is critical to chondroitin sulfate synthesis in cartilage and skeletal development in mouse and human,” *Nat. Med.*, vol. 13, no. 11, 2007.

[175] S. Tomatsu *et al.*, “Production of MPS VII mouse (Gus^TM^(hE540A.mE536ASly) doubly tolerant to human and mouse β-glucuronidase,” *Human Molecular Genetics*, vol. 12, no. 9. 2003.

[176] R. Vervoort *et al.*, “Molecular analysis of patients with β-glucuronidase deficiency presenting as hydrops fetalis or as early mucopolysaccharidosis VII,” *Am. J. Hum. Genet.*, vol. 58, no. 3, 1996.

[177] R. Vervoort *et al.*, “Molecular analysis of the β-glucuronidase gene: Novel mutations in mucopolysaccharidosis type VII and heterogeneity of the polyadenylation region,” *Hum. Genet.*, vol. 99, no. 4, 1997.

[178] E. Arikawa-Hirasawa, H. Watanabe, H. Takami, J. R. Hassell, and Y. Yamada, “Perlecan is essential for cartilage and cephalic development,” *Nat. Genet.*, vol. 23, no. 3, 1999.

[179] M. Costell *et al.*, “Perlecan maintains the integrity of cartilage and some basement membranes,” *J. Cell Biol.*, vol. 147, no. 5, 1999.

[180] K. Matsumoto *et al.*, “Conditional inactivation of Has2 reveals a crucial role for hyaluronan in skeletal growth, patterning, chondrocyte maturation and joint formation in the developing limb,” *Development*, vol. 136, no. 16, 2009.

[181] J. Lu *et al.*, “Filamin B mutations cause chondrocyte defects in skeletal development,” *Hum. Mol. Genet.*, vol. 16, no. 14, 2007.

[182] E. K. Mis, K. F. Liem, Y. Kong, N. B. Schwartz, M. Domowicz, and S. D. Weatherbee, “Forward genetics defines Xylt1 as a key, conserved regulator of early chondrocyte maturation and skeletal length,” *Dev. Biol.*, vol. 385, no. 1, 2014.

[183] B. Gwynn, K. Lueders, M. S. Sands, and E. H. Birkenmeier, “Intracisternal A-Particle Element Transposition into the Murine β-Glucuronidase Gene Correlates with Loss of Enzyme Activity: a New Model for β-Glucuronidase Deficiency in the C3H Mouse,” *Mol. Cell. Biol.*, vol. 18, no. 11, 1998.

[184] S. Tomatsu *et al.*, “Mucopolysaccharidosis type VII: Characterization of mutations and molecular heterogeneity,” *Am. J. Hum. Genet.*, vol. 48, no. 1, 1991.

[185] S. Tomatsu *et al.*, “Missense models [Gustm(E536A)Sly, Gustm(E536Q)Sly, and Gustm(L175F)Sly] of murine mucopolysaccharidosis type VII produced by targeted mutagenesis,” *Proc. Natl. Acad. Sci. U. S. A.*, vol. 99, no. 23, 2002.

[186] J. Shi *et al.*, “Membrane-type MMPs enable extracellular matrix permissiveness and mesenchymal cell proliferation during embryogenesis,” *Dev. Biol.*, vol. 313, no. 1, 2008.

[187] Z. Zhou *et al.*, “Impaired endochondral ossification and angiogenesis in mice deficient in membrane-type matrix metalloproteinase I,” *Proc. Natl. Acad. Sci. U. S. A.*, vol. 97, no. 8, 2000.

[188] K. Yashiro *et al.*, “Regulation of retinoic acid distribution is required for proximodistal patterning and outgrowth of the developing mouse limb,” *Dev. Cell*, vol. 6, no. 3, 2004.

[189] P. A. Krakowiak *et al.*, “Lathosterolosis: An inborn error of human and murine cholesterol synthesis due to lathosterol 5-desaturase deficiency,” *Human Molecular Genetics*, vol. 12, no. 13. 2003.

[190] R. Happle, I. Effendy, M. Megahed, S. J. Orlow, and W. Küster, “CHILD syndrome in a boy,” *Am. J. Med. Genet.*, vol. 62, no. 2, 1996.

[191] A. König, R. Happle, D. Bornholdt, H. Engel, and K. H. Grzeschik, “Mutations in the NSDHL gene, encoding a 3β-hydroxysteroid dehydrogenase, cause CHILD syndrome,” *Am. J. Med. Genet.*, vol. 90, no. 4, 2000.

[192] A. König *et al.*, “A novel missense mutation of NSDHL in an unusual case of CHILD syndrome showing bilateral, almost symmetric involvement,” *J. Am. Acad. Dermatol.*, vol. 46, no. 4, 2002.

[193] P. T. Clayton *et al.*, “Isolated dihydroxyacetonephosphate acyltransferase deficiency presenting with developmental delay,” *J. Inherit. Metab. Dis.*, vol. 17, no. 5, 1994.

[194] B. Itzkovitz *et al.*, “Functional characterization of novel mutations in GNPAT and AGPS, causing rhizomelic chondrodysplasia punctata (RCDP) types 2 and 3,” *Hum. Mutat.*, vol. 33, no. 1, 2012.

[195] G. Nimmo, S. Monsonego, M. Descartes, J. Franklin, S. Steinberg, and N. Braverman, “Rhizomelic chrondrodysplasia punctata type 2 resulting from paternal isodisomy of chromosome 1,” *Am. J. Med. Genet. Part A*, vol. 152, no. 7, 2010.

[196] R. Ofman, E. H. Hettema, E. M. Hogenhout, U. Caruso, A. O. Muijsers, and R. J. A. Wanders, “Acyl-CoA:Dihydroxyacetonephosphate acyltransferase: Cloning of the human cDNA and resolution of the molecular basis in rhizomelic chondrodysplasia punctata type 2,” *Hum. Mol. Genet.*, vol. 7, no. 5, 1998.

[197] C. Rodemer *et al.*, “Inactivation of ether lipid biosynthesis causes male infertility, defects in eye development and optic nerve hypoplasia in mice,” *Human Molecular Genetics*, vol. 12, no. 15. 2003.

[198] T. P. Thai *et al.*, “Impaired membrane traffic in defective ether lipid biosynthesis,” *Hum. Mol. Genet.*, vol. 10, no. 2, 2001.

[199] R. J. A. Wanders, H. Schumacher, J. Heikoop, R. B. H. Schutgens, and J. M. Tager, “Human dihydroxyacetonephosphate acyltransferase deficiency: A new peroxisomal disorder,” *J. Inherit. Metab. Dis.*, vol. 15, no. 3, 1992.

[200] Y. P. Li, W. Chen, Y. Liang, E. Li, and P. Stashenko, “Atp6i-deficient mice exhibit severe osteopetrosis due to loss of osteoclast-mediated extracellular acidification,” *Nat. Genet.*, vol. 23, no. 4, 1999.

[201] U. Kornak *et al.*, “Loss of the CIC-7 chloride channel leads to osteopetrosis in mice and man,” *Cell*, vol. 104, no. 2, 2001.

[202] A. V. Neutzsky-Wulff, M. A. Karsdal, and K. Henriksen, “Characterization of the bone phenotype in ClC-7-deficient mice,” *Calcif. Tissue Int.*, vol. 83, no. 6, 2008.

[203] N. Camacho *et al.*, “Dominant TRPV4 mutations in nonlethal and lethal metatropic dysplasia,” *Am. J. Med. Genet. Part A*, vol. 152, no. 5, 2010.

[204] M. M. Weinstein, S. W. Tompson, Y. Chen, B. Lee, and D. H. Cohn, “Mice expressing mutant Trpv4 recapitulate the human TRPV4 disorders,” *J. Bone Miner. Res.*, vol. 29, no. 8, 2014.

[205] H. I. Shin *et al.*, “gp130-Mediated Signaling Is Necessary for Normal Osteoblastic Function in Vivo and in Vitro,” *Endocrinology*, vol. 145, no. 3, 2004.

[206] T. J. Cho *et al.*, “A single recurrent mutation in the 5′-UTR of IFITM5 causes osteogenesis imperfecta type v,” *Am. J. Hum. Genet.*, vol. 91, no. 2, 2012.

[207] J. Ousingsawat, P. Wanitchakool, R. Schreiber, M. Wuelling, A. Vortkamp, and K. Kunzelmann, “Anoctamin-6 controls bone mineralization by activating the calcium transporter NCX1,” *J. Biol. Chem.*, vol. 290, no. 10, 2015.

[208] C. R. Farber *et al.*, “A novel IFITM5 mutation in severe atypical osteogenesis imperfecta type VI impairs Osteoblast production of pigment epithelium-derived factor,” *J. Bone Miner. Res.*, vol. 29, no. 6, 2014.

[209] M. Grover *et al.*, “Osteogenesis imperfecta without features of type v caused by a mutation in the IFITM5 gene,” *J. Bone Miner. Res.*, vol. 28, no. 11, 2013.

[210] E. Guillén-Navarro *et al.*, “Two mutations in IFITM5 causing distinct forms of osteogenesis imperfecta,” *Am. J. Med. Genet. Part A*, vol. 164, no. 5, 2014.

[211] F. Rauch *et al.*, “Crispr-Cas9 engineered osteogenesis imperfecta type V leads to severe skeletal deformities and perinatal lethality in mice,” *Bone*, vol. 107, 2018.

[212] O. Semler *et al.*, “A mutation in the 5′-UTR of IFITM5 creates an in-frame start codon and causes autosomal-dominant osteogenesis imperfecta type v with hyperplastic callus,” *Am. J. Hum. Genet.*, vol. 91, no. 2, 2012.

[213] J. R. Shapiro *et al.*, “Phenotypic variability of osteogenesis imperfecta type v caused by an IFITM5 mutation,” *J. Bone Miner. Res.*, vol. 28, no. 7, 2013.

[214] Y. Ju *et al.*, “Troponin T3 expression in skeletal and smooth muscle is required for growth and postnatal survival: Characterization of Tnnt3tm2a(KOMP)Wtsi mice,” *Genesis*, vol. 51, no. 9, 2013.

[215] H. W. A. Ehlen *et al.*, “Inactivation of anoctamin-6/Tmem16f, a regulator of phosphatidylserine scrambling in osteoblasts, leads to decreased mineral deposition in skeletal tissues,” *J. Bone Miner. Res.*, vol. 28, no. 2, 2013.

[216] Q. Li, Q. Lu, G. Estepa, and I. M. Verma, “Identification of 14-3-3σ mutation causing cutaneous abnormality in repeated-epilation mutant mouse,” *Proc. Natl. Acad. Sci. U. S. A.*, vol. 102, no. 44, 2005.

[217] E. Vernersson Lindahl, E. L. Garcia, and A. A. Mills, “An allelic series of Trp63 mutations defines TAp63 as a modifier of EEC syndrome,” *Am. J. Med. Genet. Part A*, vol. 161, no. 8, 2013.
